# Using 4+ to grade near-normal muscle strength does not improve agreement

**DOI:** 10.1186/s12998-017-0159-6

**Published:** 2017-10-10

**Authors:** Søren O’Neill, Sofie Louise Thomsen Jaszczak, Anne Katrine Søndergaard Steffensen, Birgit Debrabant

**Affiliations:** 1 Spinecenter of Southern Denmark, Lillebælt Hospital, Østre Hougvej 55, DK-5500 Middelfart, Denmark; 2 Institute of Regional Health Research, University of Southern Denmark, Campusvej 55, DK-5230 Odense M, Denmark; 3 Department of Sports Science and Clinical Biomechanics, University of Southern Denmark, Campusvej 55, DK-5230 Odense M, Denmark; 4 Department of Epidemiology, Biostatistics and Biodemography, University of Southern Denmark, Winsløwsvej 9, DK-5000 Odense C, Denmark

**Keywords:** Agreement, Reliability., Muscle testing., Medical Research Council scale.

## Abstract

**Background:**

Manual assessment of muscle strength is often graded using the ordinal Medical Research Council (MRC) scale. The scale has a number of inherent weaknesses, including poorly defined limits between grades ‘4’ and ‘5’ and very large differences in the span of muscle strength encompassed by each of the six grades. It is not necessarily obvious how to *convert* a manual muscle test finding into an MRC grade. Several modifications which include intermediate grades have been suggested to improve the MRC scale and the current study examines whether agreement improves and variation in ratings decrease, with an intermediate grade between ‘4’ and ‘5’, in circumstances where such a grade would seem appropriate. The present study examined the hypothesis, that a modified MRC-scale which included the commonly used ‘4+’ option, resulted in greater agreement between clinicians compared to the standard MRC-scale.

**Method:**

A questionnaire containing five simple clinical cases were distributed to a large convenience sample of chiropractors in Northern Europe, with instructions to grade the described muscle strength findings using the MRC scale. The scale was adapted (with/without an intermediate ‘4+’ grade) depending on the preference of the individual respondent. The cases were designed in such a way as to suggest a muscle weakness in the grey area between ‘4’ and ‘5’, i.e. grade ‘4+’ on the modified MRC scale.

**Results:**

A total of 225 questionnaires were returned (7% response rate). The average percentage agreement (across cases) in the standard MRC group was 64% [range 51%: 73%] (grade ‘4’ in all cases). In the modified MRC group, the corresponding findings was 48% [38%: 74%] (grade ‘4’ or ‘4+’ in all cases). The mean *average deviation analogue* in the standard MRC group was 0.34 (range 0.34: 0.40), compared to 0.51 (range 0.39: 0.73) in the modified MRC group, indicating greater dispersion of scores in the modified MRC group. The Fleiss kappa was 0.02 (*p* < 0.001) and 0.13 (*p* < 0.001), respectively.

**Conclusions:**

Contrary to the original hypothesis, introduction of a ‘4+’ grade did not clearly improve agreement or variability of ratings, despite eliminating the physical muscle testing by providing written descriptions of test findings and specifically designing these to suggest a weakness of grade ‘4+’.

**Electronic supplementary material:**

The online version of this article (10.1186/s12998-017-0159-6) contains supplementary material, which is available to authorized users.

## Background

The clinical importance of accurate and sensitive muscle strength assessment should be obvious --- in some musculoskeletal disorders (e.g. lumbar radiculopathy) the appropriate clinical course of action for a given patient, may be dictated to a large extend by the degree of muscle paresis. Many clinicians use manual muscle testing to assess muscular paresis and use the *Medical Research Council* scale of muscle strength (*MRC-scale*, also known as the Oxford scale) [1, 2] to quantify and communicate their findings. Manual and functional muscle testing procedures are quick, safe, simple to perform and require no specialist equipment. Likewise, the MRC-scale, which has been in use since the early 1940s appears simple to use and interpret, using numbers between 0 and 5 -- see Table 1.

**Table 1:** The (standard) MRC-scale of muscle strength/weakness

Grade	Description
0	No contraction
1	Flicker or trace of contraction
2	Full range of active movement, with gravity eliminated
3	Active movement against gravity
4	Active movement against gravity and resistance
5	Normal power

With sufficient training it is possible to get acceptable reliability of manual muscle testing when using the MRC-scale to grade the findings [3], but some researchers point out that functional tests of muscle strength such as e.g. heel- and toe walking, standing up from a seated position, etc. are more appropriate from a clinical point of view [4, 5]. Several studies show that quantitative muscle testing using specialist equipment such as dynamometers can identify muscle weakness which go undetected by both manual and functional muscle testing [3, 6–9] and thus the relatively poor sensitivity of manual muscle testing has prompted Bohannon [10] to cast doubt on the suitability of such procedures as a screening tool.

In addition to the potential short-comings of manual muscle testing procedures, there are also a number of caveats to bear in mind regarding the appropriateness of the MRC-scale itself. In fact, when used in conjunction, it becomes difficult to disentangle whether such short-comings stem from the test procedures themselves or from the process of converting test findings to a grade on the MRC scale.

The use of the numbers 0 to 5 for the MRC-grades is unfortunate, as it suggests equidistant points along a continuous scale -- the kind of scale which would be appropriate for *measurement* of muscle strength using a dynamometer. In reality however, the numbers 0 to 5 on the MRC-scale simply represent *grades* or ordered steps on an ordinal scale (see info Table 2 for further details). Deep tendon reflexes may be graded using a similar scale [11], which could be a source of confusion.

**Table 2:** The MRC grades 0-5 represent *ordinal* data

The *ordinal* data type is a sub-group of the *categorical* data type, in which different categories are *ordered* in relation to each other. An example of ordinal data could be a measure of experience, on a scale with three *ordered* categories: a) novice, b) experienced and c) expert. The MRC-scale is also an ordinal data type and the grade ‘4’ for instance, represents a muscle strength greater than ‘3’ and less than ‘5’. Confusingly, the difference in numerical value is 1 in both directions, but the *step up* from ‘3’ to ‘4’ is not necessarily the same as the step up from ‘4’ to ‘5’ in the same way that the difference between ‘novice’ and ‘experienced’ is not necessarily the same as that between ‘experienced’ and ‘expert’. Neither does a grade ‘2’ on the MRC-scale necessarily represent a meaningful mean value of ‘1’ and ‘3’. As such, the numbers only represent *designations* or *names* of categories on an ordinal scale and arguably it would have been less confusing to designate the steps on the scale in some other way, e.g. using letters A to F.	

Furthermore, the grades ‘0’, ‘1’, ‘2’ and ‘3’ represent muscle strength/weakness with objectively verifiable cut-off points. For example, either visible signs of muscle contraction are present (grade 1) or they are not (grade 0). Conversely, grades ‘4’ and ‘5’ are subjective in nature, relying not only on a clinical examination procedure, but also a subjective clinical judgement of what constitutes ‘*normal*’ or ‘*weak*’ muscle strength.

Also, each grade represents hugely different intervals. Arguably grade ‘5’ represents only a single end-point on the scale, namely *normal muscle strength*, and anything less than normal should be graded as 4 or less. A grade of ‘4’ by contrast, encompasses a very large span of muscle weakness: From just below normal to considerable weakness with significant functional disability. Grade ‘4’ may in fact represent as much as 96% of the total spectrum of muscle strength [12] and Dvir [13] concludes that: “*It may therefore be stated that the application of grade 4 [...] is not valid where an accurate figure relating to muscle strength deficiency is required.*”. In other words, the grade ‘4’ may encompass a span of weakness so large that clinically relevant details are lost.

When researchers have reported good reliability with manual muscle tests, this may in part reflect a poor discriminatory ability of the MRC-scale rather than good reliability of the testing procedure itself -- if a single category on the scale occupies more than 90% of the entire scale spectrum, reliability is bound to be good. Mahony et al. [8] for example, reported fair to excellent reliability with manual muscle testing and reported the *minimum detectable change* to be one scale grade on the MRC-scale. It is worth contemplating, what a minimum detectable change of one grade means when grade ‘4’ occupies almost the entire spectrum of sub-normal muscle strength.

Finally, choosing between grades ‘4’ and ‘5’ can also be unclear in relation to *apparent* muscle weakness: Which grade is appropriate for a grade ‘4’ weakness deemed to stem, not from physiological paresis as such, but from pain provocation, poor compliance, exaggeration, etc.? Some clinicians may choose to grade such an apparent weakness as ‘5’. Other clinicians may chose to grade it as ‘4’ and (hopefully) add comments to elaborate on their clinical judgement. The ambiguous descriptors at the upper end of the MRC-scale do not offer much to guide the clinician in this respect.

With the above mentioned caveats in mind, the distinction between grades ‘4’ and ‘5’ on the MRC-scale is thus likely to be particularly difficult and the study by Paternostro-Sluka et al. confirm this [14]. The authors examined 31 patients with more than 3 months of weakness in the myotome of the radial nerve and identified a sub-group of patients (*n* = 20) which were deemed to have normal grip strength *by manual muscle testing*. When measured using a dynamometer however, the authors reported a median grip strength of 65% on the affected side, compared to the unaffected side. In other words, a considerable weakness on the affected side went undetected by manual muscle testing. Findings to the same effect are reported by Rabin and Post [7], Bohannon [10] and Ustun et al. [9].

It may be the case, that manual muscle testing is simply not sensitive enough to detect even manifest muscle weakness. At face-value, findings such as those by Paternostro-Sluka et al., certainly appear to suggest so. However, there is another plausible explanation: It is possible that manual muscle testing is actually able to discriminate smaller differences in weakness, than the crude categories of the MRC-scale will allow for. In other words, that the finer details of manual muscle testing is simply lost when converted to the MRC-scale. Whether this is actually the case is unclear, but it would appear to be the rationale for introducing intermediate grades such as ‘4+’.

In fact, several modifications and alternatives to the MRC-scale have been suggested over the years, which provide finer-grained categorization of test findings. One such modification was suggested by the Medical Research Council itself, in the 1974 follow up to the original publication [1]: "*Grades 4-, 4 and 4+, may be used to indicate movement against slight, moderate and strong resistance respectively*". However, a distinction between ‘slight’, ‘moderate’ or ‘strong’ resistance was not defined and as such the modification would only introduce an extra level of subjectiveness to the grading. Barr et al. [15] suggested adding 3-, 3+, 4- and 4+ to the scale. Paternostro-Sluka et al. [14] suggested introducing intermediate grades for 2–3, 3–4 and 4–5 and Bohannon [10] suggested a comprehensive modification of the MRC scale with a total of 12 grades.

In summary therefore, previous findings suggest that manual muscle testing graded by the MRC-scale has relatively poor sensitivity compared to objective measurement of muscle strength and that even pronounced muscle weakness can go undetected. Paradoxically however, researchers have apparently considered the 6 categories of the original MRC-scale to have too low discriminatory ability to capture the finer details of manual muscle testing, which could be interpreted to mean that the problem is not with clinical muscle testing itself, but rather the categorization provided by the MRC-scale. And this would appear to be particularly pronounced in the *grey area* between grades ‘4’ and ‘5’. Furthermore, in the context of spinal pain and radiculopathy, the clinical experience is, that muscle weakness of grades ‘0’-to-'3′ is rare and it is our clear impression that the most commonly used modification to the original MRC-scale is adding ‘4+’ as an intermediate step between ‘4’ and ‘5’.

The aim of the present study was to determine whether a modified MRC-scale which included the commonly used ‘4+’ option, resulted in greater agreement between clinicians compared to the standard MRC-scale --- specifically in circumstances where ‘4+’ would appear to be the correct grade and where the variability from actual physical muscle testing procedures could be disregarded. The hypothesis was, that respondents using the *modified* MRC-scale would choose ‘4+’ predominantly and thus have a higher degree of agreement compared to respondents using the *standard* MRC-scale.

## Methods

A questionnaire was constructed by the authors (principally SON) and distributed electronically to a convenience sample of relevant clinicians in Denmark, Norway, Sweden, Britain and Germany. The questionnaire and translations were not validated independently, but consisted of 5 simple clinical cases which included a description of muscle strength. The respondents were asked to grade the described muscle strength using either the MRC-scale or a modified MRC-scale, in accordance with their personal preference. Each case was presented separately with an accompanying MRC scale and the questionnaire could be completed in less than 10 min.

### Participants

Relevant professional associations (chiropractic, medicine and physiotherapy) in Denmark, Norway, Sweden and Germany, as well as the British Chiropractic Association were approached and asked to distribute the questionnaire electronically to their members. For details, please see the Additional file 1.

The professional associations were asked to distribute questionnaire links to their members and data were collected between March 3rd and April 16th 2016. No reminders were sent.

### Questionnaire

In the first page of the questionnaire, participants were instructed to relate the subsequent cases to that of a ‘*classical*’ lumbar disk herniation patient with low-back pain, unilateral sciatica and an MRI-confirmed disk herniation at e.g. L5-S1. It was explained that the questionnaire was not a test of diagnostic skills, but rather how clinicians interpret muscle weakness and make use of the 0-to-5 MRC-scale. At the end of the first page, participants were asked: “*Besides the values 0 to 5 in the grading, some clinicians use the value of 4+ or 4½ in the journal, when they believe it is relevant. Do you do that? (yes/no)*”.

Following this first introductory page, participants were presented with 5 short clinical cases, each of which described a degree of paresis on muscle testing. The descriptions were deliberately worded in such a way as to be ambiguous about which category of the standard MRC scale was appropriate. E.g. the first question read: "*When testing the patient, you find good muscle strength at plantar flexion on the affected side and estimate it as normal, but you estimate less muscle strength than on the not-affected side.*". The grading of the case is ambiguous in so far as the muscle strength is described both as ‘*normal*’ and as ‘*less [..] than the not-affected side*’, which arguably should be graded as ‘5’ and ‘4’ on the standard MRC-scale, respectively. See Additional file 1 for the wording of all 5 cases in each of the provided languages.

The same five cases were presented to all participants, but two different scales (standard or modified MRC-scales) were provided for participants to grade the described muscle strength. Participants who had indicated using ‘4+’ or ‘4½’ in their journals (hereafter the *modified MRC*-group) were presented with a modified MRC-scale (0, 1, 2, 3, 4, 4+, 5), and those who had not, were presented with a standard MRC-scale (0, 1, 2, 3, 4, 5) (hereafter the *standard MRC*-group). In each of the five cases, we thus expected the modified MRC-group to choose the ‘4+’ option, and the standard MRC-group to choose either ‘4’ or ‘5’.

For each case, a short free-text field was provided allowing the participants to elaborate on their answers.

To keep the length of the questionnaire to a minimum, no other data were collected.

### Data and statistical analysis

The questionnaire was constructed using SurveyXact (v12.4) and data were analysed using R (v3.3.1, x86_64 for Linux). A response rate was calculated as the percentage responses of the potential *maximum* number of respondents. The main results are presented as *average deviation analogue* (ada) as described by Wilcox [16] and *percentage agreement*, i.e. the frequency of the most common answer, as recommended for ordinal data by de Vet et al. [17]. The distribution of grades is illustrated graphically using mosaic plots as recommended by Friendly [18].

The *average deviation analogue* is “an analogue of the average or mean deviation” for categorical data. The *ada* statistic produces values between 0 and 1, where 0 represents a situation in which there is no deviation -- all observations are confined to only one of the possible answers (categories). Conversely, an *ada* of 1 represents a situation in which observations are distributed evenly between all possible answers [16].

Further, to assess agreement, we applied the kappa statistic as described in Landis and Koch “A one-way components of variance model for categorical data.” [19].

## Results

The chiropractic associations in Denmark, Norway, Sweden, Great Britain and Germany and the medical association in Schleswig-Holstein (the northernmost German state) distributed the questionnaire to their members. The remaining organizations either rejected or ignored the request. According to the membership numbers listed on the association websites (see Table 3), the questionnaire could potentially have been distributed to a maximum of 3378 association members, 3328 of which were chiropractors. However, we have no means of verifying exactly how many invitations were actually distributed.

**Table 3:** Membership numbers of participating professional organizations

Association	Members
Danish Chiropractic Association	874
Norwegian Chiropractic Association	784
Swedish Chiropractic Association	200
British Chiropractic Association	1350

Membership numbers based on organizational web pages at the time of the study

Inaccuracies in the German translation were identified after data collection and only 14 German questionnaires were returned -- These have been excluded from analysis.

**Fig. 1:**
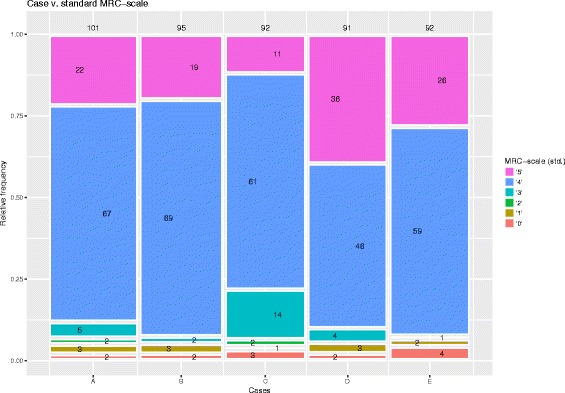
Frequency of scores by question -- standard MRC scale. Mosaic-plot of the relative frequency of answers zero-to-five for cases A-E in the *standard MRC group*. Numbers in boxes represent absolute frequency. Numbers over each column is total number of answers for that case

**Fig. 2:**
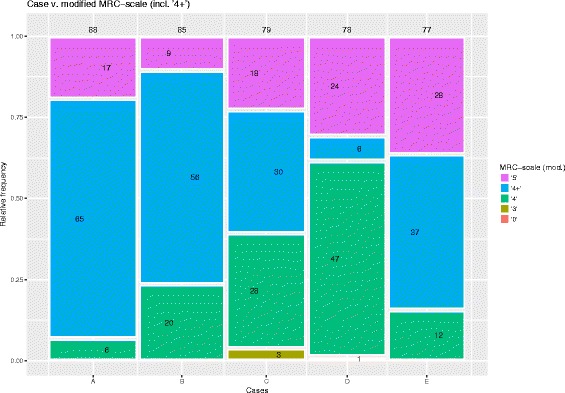
Frequency of scores by question -- modified MRC scale. Mosaic-plot of the relative frequency of answers zero-to-five for cases A-E in the modified MRC group. Numbers in boxes represent absolute frequency. Numbers over each column is total number of answers for that case

Of the 225 respondents (7% response rate), 131 indicated using the standard MRC-scale (standard MRC-group), of which 77 completed all 5 cases. 94 respondents indicated using the ‘4+’ grade (modified MRC-group) of which 91 completed all 5 cases. Nationality and MRC-group is summarized in Table 4. All submitted responses are illustrated graphically in Figs. 1 and 2 -- subdivided into the standard MRC and modified MRC groups, respectively.

**Table 4:** Summary of MRC group and nationality

	Danish	English	Norwegian	Swedish	Total
Modified	31	17	36	10	94
Standard	38	7	44	42	131
Total	69	24	80	52	225

*Modified* and *Standard* refers to participants using a modified MRC scale (including a ‘4+’ option) and those using the standard MRC scale

It is evident that most cases were graded as either ‘5’, ‘4+’ or ‘4’ as expected, but also that 6.7% were graded as ‘3’ or less. Examining the free-text fields related to grades ‘3’ and less, revealed that a number of respondents reported being unfamiliar with the MRC-scale and/or had apparently turned the scale up-side-down, grading all five cases as ‘0’ or ‘1’. This is discussed in the following section, but for completeness, results are presented both with and without responses of ‘3’ and less. In retrospect, it would been advantageous to include a question on whether or not respondents made use of the MRC scale at all.

Table 5 lists the percentage agreement and average deviation analogue for each case, by group.

**Table 5:** Percentage agreement and average deviation analogue

Case	Standard	Modified	Standard..limited.	Modified..limited.
A	66% [0.34]	74% [0.39]	75% [0.49]	74% [0.39]
B	73% [0.34]	66% [0.51]	78% [0.43]	66% [0.51]
C	66% [0.4]	38% [0.69]	85% [0.31]	39% [0.86]
D	51% [0.37]	60% [0.45]	56% [0.88]	61% [0.58]
E	64% [0.35]	48% [0.73]	69% [0.61]	48% [0.73]
ALL	64% [0.34]	48% [0.51]	73% [0.55]	48% [0.78]

The percentage of the most common answer and [average deviation analogue] for each case in the standard and modified MRC groups (limited and not limited to ‘*correct*’ answers greater than 3). Higher percentages indicate a greater agreement between raters. Higher *ada* values indicate greater (qualitative) variation

Example: Fig. 1 illustrates that ‘4’ was the most common answer (*n* = 67) in case A in the standard MRC group. This constitutes 66% (Table 5) of the total case A responses in that group (*n* = 101 – Fig. 1, above column A). Excluding the case A responses graded as ‘3’ or less (*n* = 5 + 2 + 3 + 2 -- see Fig. 1), this changes to 75% (Table 5).

In the standard MRC group, grade ‘4’ was the most common choice in all 5 cases (see Fig. 1). In the modified MRC group the most common answer was either ‘4+’ (cases A, B, C and E) or ‘4’ (case D) (see Fig. 2).

The kappa was 0.02 (*p* < 0.001) and 0.13 (*p* < 0.001) for the standard and modified MRC groups, respectively. When limited to 3+ answers, the results were 0.06 (*p* < 0.001) and 0.14 (*p* < 0.001).

## Discussion

This study aimed at examining, whether extending the standard MRC scale to a modified MRC scale improves agreement and reduces variation of the ratings.

Overall, the data does not demonstrate a greater percentage-agreement in the modified MRC group compared to the standard MRC group and visual inspection of Figs. 1 and 2 suggest that if anything there is greater variation between grades and between cases in the modified MRC group.

The average deviation analogue was also found to be greater in nine of ten comparisons (all five cases, limited and not limited) in the modified MRC group compared to the standard MRC group, as well as for all cases combined, indicating that ratings were more evenly distributed and less focused in the modified MRC group.

Kappa values for both standard and modified MRC scales were very low (0.05 resp. 0.14). Although, for both scales the hypothesis of no agreement exceeding pure chance agreement (kappa = 0) was rejected with *p* < 0.0001, there seemed to be a rather large amount of variability in the given ratings and the strength of agreement is poor for both rater groups.

Caution should be exercised, when drawing conclusions from the observed percentage agreement, average deviation analogue and kappa due to the fact that the number of categories is increased by one when switching from the standard MRC to the modified MRC scale. For example, with an increase from 2 to 3 categories on a given scale, the *minimum* possible percentage agreement that can be observed drops from 50% to 33.3%. The ada index ranging from 0 to 1 overcomes this problem in that its maximum and minimum values do not depend on the number of categories. Intermediate ada indices however, typically decrease when increasing the number of categories into which cases are rated, not necessarily preserving the order of the cases when ordered according to their ada indices (refer to Wilcox “Indices of qualitative variation ...” for an illustrative example) [16].

In other words, with more categories available to chose from, the agreement is likely to decrease (and dispersion to increase), at least if the entire span of the scale is used. It should be noted however, that the current questionnaire was intentionally designed to focus on a very limited part of the scale: with 2 *correct* answers (grades ‘4’ and ‘5’) on the standard MRC scale and only one (‘4+’) on the modified MRC scale. Thus, *if* a greater agreement and lower dispersion had indeed been found in the modified MRC group as hypothesized, this could to some extend have been the result of more (2 vs 1) correct answer options on the standard MRC scale. But as described, the current findings suggest lower agreement and greater dispersion in the modified MRC group, despite the effects of differences in number of categories. In the section below we discuss whether the five cases were in fact successfully constructed in such a way as to focus on answers of ‘4’ or ‘5’ on the standard MRC scale and ‘4+’ on the modified scale.

Finally, kappa is a measure of agreement usually used in studies with a relatively large number of cases and a relatively small number of raters. It *can* be applied to situations with a low number of cases assessed by a larger number of raters, but will typically result in relatively low kappa values. Irrespective thereof, the expectation would still be to see a decrease in kappa when increasing the number of categories, i.e. going from standard MRC to modified MRC (see eg. Altmann “Practical Statistics for Medical Research”) [20].

In summary, the agreement is poor for both scales. The fact that we observed an increase in kappa, when using the modified instead of the standard MRC scale, could in principle be taken as an indication of slightly increased agreement of the modified MRC scale. However this cannot be tested rigorously, since standard errors for the observed change in kappa values are not available in the given context. By contrast, an increase in ada for the modified MRC scale indicates a greater qualitative variation in ratings, when compared to the standard MRC and thus less consensus among those raters. Observing the percentage agreement decreasing in most of the cases as well as for all cases combined, does not provide further indication of improved agreement and conclusions thus remain ambivalent. Altogether, the data does not suggest a clear improvement in agreement using the modified MRC scale.

### Case descriptions

With cases specifically designed to describe muscle weakness *ambiguously* in the grey area between ‘4’ and ‘5’ on the standard MRC-scale, it was to be expected that raters in the standard MRC group would be uncertain about which of those two options to choose. Conversely, raters in the modified MRC group could resolve such uncertainty by choosing the ‘*middle*’ value of ‘4+’. This in turn was expected to result in greater agreement and less variability in ratings in the modified MRC group compared to the standard MRC group. As concluded, this was not found to be the case.

An ambiguously described weakness *somewhere between* ‘4’ and ‘5’ should not be expected to result in a equal distribution of ‘4’ and ‘5’ grades in the standard MRC group and it is reasonable to expect clinicians to place greater emphasis on the abnormal findings in the case description, than on the normal findings. Indeed grade ‘4’ was roughly 3 three times more common than ‘5’ in the standard MRC group.

Apart from case C, responses of ‘3’ or less in the standard MRC group can generally be ascribed to participants unfamiliar with the MRC scale and/or participants having turned the scale upside-down, scoring most cases as 0, 1 or 2. The higher frequency of answers of ‘3’ and less in the standard MRC group, is explained by the wording of question #1. Participants unfamiliar with the MRC scale would naturally answer ‘No’ to question #1, thus placing them in the standard MRC group. Raters unfamiliar with the MRC scale would thus predominantly be found in the standard MRC group. To counter this, we have presented results based on all the available data (‘unlimited’) as well as data limited to appropriate answers at the upper end of the scale.

The distribution of responses in case A was essentially similar between groups, with ‘4+’ simply replacing ‘4’ in the modified MRC group. In case B however, a proportionally larger number of raters in the modified MRC group chose ‘4’, despite having the ‘4+’ option available to them. As described in the section above, some raters chose ‘3’ in case C -- not because of unfamiliarity with the scale, but because they felt weakness against gravity and gradually increasing weakness warranted a grade ‘3’. In any case, the vast majority of raters in the standard MRC group elected ‘4’ in case C, whereas the modified MRC group was spread more uniformly over ‘4’, ‘4+’ and ‘5’, with only 3 raters choosing ‘3’. Case D was distinct from the other cases in that the answer was given in the case description: ‘*You find a weakness [..] grade 4 [..]*’, and furthermore the character of the weakness was given as ‘*mainly pain-related*’. Interestingly, 44% of the standard MRC group and 31% of the modified MRC group, still graded the case as ‘5’. In other words, a considerable number of raters elected to *translate* a weakness of grade ‘4’ into grade ‘5’ when informed that the nature of the weakness was pain related (as opposed to neuro-muscular). The standard MRC group was thus almost evenly split between grades ‘5’ and ‘4’. The modified MRC group on the other hand, chose the expected answer of ‘4+’ in only 9% of the answers (the lowest in any of the cases) and answered ‘4’ in 60% of responses. It could be argued, that a high rate of grade ‘4’ responses is unsurprising, as the case description specifically suggested that a grade 4 was observed. However, both groups had little reservation in choosing ‘5’ despite the case description and furthermore, the ‘4+’ option available to the modified MRC group would also be in line with the case description and could serve as a way to qualify a grade ‘4’ weakness as pain-related -- yet it was the least common answer in the modified MRC group (bar the 2 answers of ‘0’). The availability of a ‘4+’ option apparently did not tempt the modified MRC group to choose that grade as a means of *qualifying* the weakness as being pain-related. Even though case E had the least convincing description of weakness, 15% of the modified MRC group still chose ‘4’ -- again the availability of a ‘4+’ option did not sway them, nor improve agreement.

Overall thus, we would argue that the five cases were ambivalently worded in such a way as to favor a ‘*middle*’ response in the grey area between ‘4’ and ‘5’. Accepting this premise, the results should arguably have been greater agreement in the modified MRC group, compared to the standard MRC group. Instead, the ‘4+’ option apparently only added an extra answer option to be ambivalent about, resulting in greater, rather than less disparity in the modified MRC group.

The current study only examined the effect of adding ‘4+’ to the MRC-scale, as that end of the scale would appear to be the most difficult to grade. Based on the current data we cannot say whether the more extensively modified scales suggested by the Medical Research Council [1], Barr et al. [15], Paternostro-Sluka et al. [14] or Bohannon [10] would result in greater agreement, but it seems improbable as an even greater number of grades without clear definitions, will most likely only result in even greater dispersion. It is entirely possible of course, that agreement would improve rather than deteriorate with the addition of intermediate grades such as ‘4+’, if such grades were clearly defined with objective limits -- that is not the case however.

The current findings are not based on actual, physical examinations of muscle weakness and were not intended to examine the validity of manual muscle testing as such. Instead, the written description of clinical findings which was presented to participants, mean that variability due to different examination techniques and bias related to patient compliance, etc. was eliminated. To our knowledge, no comparable questionnaire studies have been presented previously. All raters were presented with the same clinical findings (albeit in their native language). Variability in the present data will thus predominantly reflect variance related to differences between the scales, variation between raters and possibly language-related differences in interpretation of the written case descriptions. Based on the current data, we can not disentagle these effects, but while we suspect minor language-related differences in interpretation of the questions to have little effect, it is quite possible that systematic differences between raters other than those relating to the MRC scale, is skewed between groups -- e.g. that raters unfamiliar with the MRC scale tended to answer ‘No’ to question #1.

### Study limitations

The questionnaire could potentially have been distributed to a very large number of clinicians from different professions in northern Europe. As it were, the data reflect only chiropractors and it is possible that a broader sampling of clinicians could have yielded other results. We have no particular reason to believe so however, as the MRC-scale is not specific to any one profession and is part of many common pre-graduate textbooks and clinical guidelines.

Also, to restrict the questionnaire length to a bare minimum (to increase the likelihood of participants completing it) we did not inquire about such baseline characteristics as age, sex, years in practice, main clinical interests, university/college of training, etc. It is possible, that such data could have thrown up interesting associations/contingencies and certainly it could have described the study population in greater detail. As it is, the study population can only really be described as *Northeuropean chiropractors*.

As mentioned above, it would have in retrospect have been useful to include a question about whether the respondents were familiar with the MRC at all. As a consequence, respondents unfamiliar with the scale who completed it anyway, will by default have been grouped in the standard MRC group.

## Conclusion

The current findings suggest that, irrespective of the validity and reproducibility of clinical muscle strength tests, there are separate issues relating to the MRC-scale itself. Grade ‘4’ spans a very wide interval of the muscle strength/weakness spectrum, with poorly defined criteria. But simply introducing an intermediate grade like ‘4+’ appears only to compound the issue. Most likely because the criteria for grade ‘4+’ are equally ill-defined, thus simply introducing more options to choose from, without clarifying the distinctions between them.

We would therefor draw attention to the following three points:

Any future attempts to modify the MRC scale should include specific and objectively verifiable criteria to guide the clinician -- simply adding more diffuse categories is unlikely to increase the usefulness of the scale.

From a clinical perspective, the ability to identify clinically relevant categories such as ‘*pain-related weakness*’, ‘*weakness with functional deficit*’ and ‘*weakness only on repeated loading (fatigue)*’ may be more important than poorly defined grades on an ordinal scale. Such clinically relevant categories can have direct impact on clinical decision making and we would therefor suggest, that any muscle strength assessment graded between ‘3’ and ‘5’ on the MRC scale (modified or not) be qualified with a suitable comment. It is worth noting, that a substantial number of participants in the current study made written remarks to that effect, e.g. “*I would grade it as '4', but describe that the weakness was probably due to pain*”.

From a research perspective, the categories of a clinical muscle strength scale should correspond to the discriminatory ability of muscle testing procedures. When clinically relevant details such as those described above, appear to be lost in translation it is tempting to add more categories. However, there is little sense in constructing scales with clinically relevant categories, if the examination procedures are unable to discriminate between them. The discriminatory ability of clinical muscle strength tests therefor need to be established before relevant scales can be constructed to reflect such findings.

## Additional file

Additional file 1: Questionnaire text in the provided languages, and list of professional associations [21–33]. (DOCX 8 kb)

## Abbreviations

ADA: Average Deviation Analogue
MRC: Medical Research Council
