# Supplementary material for: Using 4+ to grade near-normal muscle strength does not improve agreement
Source: Chiropr Man Therap. 2017 Oct 10;25:28. doi: 10.1186/s12998-017-0159-6 (PMC5633899; doi:10.1186/s12998-017-0159-6)
Supplement: Additional file 1: — Questionnaire text in the provided languages, and list of professional associations [21–33]. (DOCX 8 kb) [file 12998_2017_159_MOESM1_ESM.docx]

# Appendix

## Danish questionnaire text

1. Der er kun 6 spørgsmål i alt i dette skema, som er fordelt på 6 sider.Du skal forestille dig en ’klassisk’ prolaps patient med rygsmerter og unilateral iskias gennem nogle måneder og en flot prolaps på MR skanning, f. eks. på L5-S1 niveau. Det er altså ikke diagnostikken der er en udfordring og det er heller ikke en test af om man kan huske hvordan skalaen er skruet sammen. Spørgsmålene er alle relateret til hvordan vi tolker muskelsvækkelse og bruger 0-5 muskelkraft skalaen for en sådan patient, i de tilfælde hvor det (måske?) ikke umiddelbart er oplagt. Foruden værdierne 0 til 5, bruger nogle klinikere at angive muskelkraft som 4+ eller 4½ i journalen, når de mener det er relevant.
2. Du finder god muskelkraft ved plantar-fleksion på den afficerede side, og ville umiddelbart vurdere den som normal, men den er dog mindre end på den ikke-afficerede side. Hvorledes vil du i så fald ’score’ muskelkraften?
3. Du finder en lettere svækkelse af plantar-fleksionen ved manuel test. Samtidig er tå-gang dog normal. Hvorledes vil du i så fald ’score’ muskelkraften? 4.Du finder normal muskelkraft ved manuel test, rygliggende på us-lejet, men patienten har svækkelse ved mere end 5 skridts tå-gang. Hvorledes vil du i så fald ’score’ muskelkraften?
4. Du finder en muskelkraft svækkelse grad 4, men det er dit indtryk at den i betydelig grad er smertebetinget. Hvorledes vil du i så fald ’score’ muskelkraften?
5. Du finder at både manuel test og tå-gang umiddelbart er normale, men kan udtrættes ved f. eks. tå-gang over 10 skridt. Hvorledes vil du i så fald ’score’ muskelkraften?

## English questionnaire text

1. There are only 6 questions in this survey, distributed on 6 pages. Imagine a 'classic' disc herniation patient with back pain and unilateral leg pain. The pain has been present for a couple of months and the MRI shows a big disk herniation, e.g. on level L5-S1. This survey is therefore not about the diagnostic challenge and not a test to see if you remember how the scale works. All the questions are related to how we interpret muscle weakness and use the 0-5 muscle strength scale for such a patient, in cases where it (maybe?) is not obvious. Besides the values 0 to 5 in the grading, some clinicians use the value of 4+ or 4½ in the journal, when they believe it is relevant. Do you do that? (yes/no)
2. When testing the patient, you find good muscle strength at plantar flexion on the affected side and estimate it as normal, but you estimate less muscle strength than on the not-affected site. In this case, how will you rank the muscle strength?
3. You find a slight weakness of the plantar flexion when you test manually. Though, at the same time walking on toes is normal. In this case, how will you rank the muscle strength?
4. You find normal muscle strength when you test manually and the patient lies supine, but when the patient walks more than 5 steps on the toes, you estimate some weakness. In this case, how will you rank the muscle strength?
5. You find a weakness in the muscle strength grade 4 on the scale, but it is your impression that it is mainly pain-related. In this case, how will you rank the muscle strength?
6. You find that both manuel muscle strength test and toe-walk are normal, but can be exhausted by ex. toe-walk more then 10 steps. In this case, how will you rank the muscle strength?

## Norwegian questionnaire text

1. Det er bare seks spørsmål i denne undersøkelsen, fordelt på 6 sider. Tenk deg en "klassisk" diskusprolaps pasient med ryggsmerter og unilateral smerte i bena. Smerten har vært til stede i et par måneder, og MR viser en stor diskusprolaps , f.eks på nivå L5-S1. Denne undersøkelsen er derfor ikke om diagnostisk utfordring og ikke en test for å se om du husker hvordan det fungerer skalaen. Alle spørsmålene er knyttet til hvordan vi tolker muskelsvakhet og bruker 0-5 muskelstyrke skala for en slik pasient, i tilfeller der det (kanskje?) er ikke opplagt. Foruten de verdiene 0 til 5 i gradering, noen klinikere bruke verdien av 4+ eller 4½ i tidsskriftet, da de mener det er relevant.
2. Når du skal teste pasienten, finner du god muskelstyrke på plantar fleksjon på den berørte side og regner det som vanlig, men du anslå mindre muskelstyrke enn på ikke-berørte området. I dette tilfellet, hvordan vil du rangere muskelstyrke?
3. Du finner en liten svakhet i plantar fleksjon når du tester manuelt. Skjønt, samtidig er tå gange normalt. I dette tilfellet, hvordan vil du rangere muskelstyrke?
4. Du finner normal muskelstyrke nar du teste manuelt og pasienten ligger på ryggen, men när pasienten går mer enn 5 trinn på tærne, anslår deg noen svakhet. I dette tilfellet, hvordan vil du rangere muskelstyrke ?
5. Du finner en svakhet i muskelstyrke til grad 4 på skalaen, men det er ditt inntrykk at det hovedsakelig er smerte-relatert. I dette tilfellet, hvordan vil du rangere muskelstyrke?
6. Du finner ut at både manuell muskelstyrke test og tå gange er normalt, men kan bli utmattet av ex. tå gange mer enn 10 trinn. I dette tilfellet, hvor man vil rangere muskelstyrke?

## Swedish questionnaire text

1. Det är endast 6 frågor totalt i detta schema, fördelat på 6 sidor. Du ska föreställa dig en ’klassisk’ diskbråckpatient med ryggsmärtor och unilateral ischias genom några månader och en tydlig prolaps på MR scanning, t.ex. på L5-S1 nivå. Det är alltså inte diagnostiken som är en utmaning och heller inte ett test för om man kommer ihåg hur skalan är uppbyggd.Alla frågor relaterar till hur vi tolkar muskelförsvagning och hur 0-5 muskelkraft-skalan används för en sådan patient i de tillfällen där det (kanske?) omedelbart inte är uppenbart. Utöver graderna 0 till 5 väljer vissa kliniker att ange muskelkraft som 4+ eller 4½ i journalen, när det tycks relevant.
2. Du hittar en god muskelkraft för plantarflexion på den påverkade sidan och tycker omedelbart den är normal. Dock är kraften mindre än på den motsatta sidan. Hur bedömer du i detta tillfälle muskelkraften?
3. Du hittar en lätt muskelförsvagning för plantarflexion i din undersökning. Samtidigt är tågången dock normal. Hur bedömer du i detta tillfälle muskelkraften?
4. Du hittar en normal muskelkraft i din undersökning med patienten liggandes på rygg, men patienten har muskelförsvagning vid mer än 5 steg vid tågång. Hur bedömer du i detta tillfälle muskelkraften?
5. Du hittar en muskelförsvagning grad 4, men du tycker att det i stor grad är smärtrelaterat. Hur bedömer du i detta tillfälle muskelkraften?
6. Du finner att både undersökning av muskelkraft och tågång är normal, men kan uttröttas med t.ex. tågång över 10 steg. Hur bedömer du i detta tillfälle muskelkraften?

## Professional associations

- Dansk Kiropraktor Forening (Danish Chiropractors' Association[21])
- Lægeforeningen, Danmark (Danish Medical Association[22])
- Danske fysioterapeuter (The Association of Danish Physiotherapists[23])
- British Chiropractic Association[24]
- Legitimerade Kiropraktorers Riksorganisation (Swedish Chiropractic Association[25])
- Sveriges läkarförbund (Swedish Medical Association[26])
- Fysioterapeuterna Sverige (Swedish Association of Physiotherapists[27])
- Norsk Kiropraktorforening (Norwegian Chiropractic Association[28])
- Den Norske Legeforening (The Norwegian Medical Association[29])
- Norsk Fysioterapeutforbund (Norwegian Physiotherapist Association[30])
- German Chiropractors Association (German Chiropractors Association[31])
- Deutscher Verband für Physiotherapie (German Association of Physiotherapists[32])
- Bundesärztekammer Deutschland (German Medical Association[33])
